# Optoelectronic Characterization of Trap Density of States in Indium Gallium Oxide Thin-Film Transistors and Their Impact on Bias Stability

**DOI:** 10.1021/acsami.5c21764

**Published:** 2026-02-19

**Authors:** Sang Yeon Kim, Je-Jun Lee, Jae Seok Hur, Buyeon Kim, Jung Pyo Hong, Seong-Jun Han, Eungseon Yeon, Jung Woo Kim, Jae Kyeong Jeong, Do Kyung Hwang

**Affiliations:** † Center of Quantum Technology, Post-Silicon Semiconductor Institute, 58975 Korea Institute of Science and Technology (KIST), Seoul 02792, Republic of Korea; ‡ Department of Electronic Engineering, 26716 Hanyang University, Seoul 04763, Republic of Korea; § KU-KIST Graduate School of Converging Science and Technology, Korea University, Seoul 02841, Republic of Korea; ∥ Division of Nanoscience & Technology, KIST School, University of Science and Technology (UST), Seoul 02792, Republic of Korea

**Keywords:** crystalline oxide semiconductor thin-film transistor, indium gallium oxide, trap density of states, photoexcited charge collection spectroscopy, photoresponse capacitance–voltage, bias stress stability

## Abstract

Amorphous oxide semiconductors have become the industry standard for display backplanes, but their limited mobility necessitates complex low-temperature polycrystalline oxide (LTPO) stacks, increasing cost and reducing yield. To realize a practical oxide-only backplane that can serve as both drive and switching transistors, a channel combining high mobility, low off current, and robust stability is required. Here, we report zinc-free crystalline indium–gallium oxide (IGO) thin-film transistors (TFTs) that crystallize below 400 °C with preferential (222) orientation. By tuning the In:Ga ratio, an amorphous-to-crystalline transition is achieved, enhancing mobility to 83.3 cm^2^ V^–1^ s^–1^ while maintaining an off-current of ≈10^–13^ A. The optimized crystalline IGO TFTs (In:Ga = 12:3) exhibit the smallest threshold-voltage shift (<0.5 V) under bias stress and reproducible electrical characteristics across multiple devices. Furthermore, we provide experimental investigation of the observed stability by analyzing the trap density of states (TDOS) near both the valence-band maximum (VBM) and conduction-band minimum (CBM) using a combined optoelectronic approach based on photoexcited charge collection spectroscopy (PECCS) and photoresponse capacitance–voltage (C–V). The complementary analysis reveals that crystalline IGO stabilizes oxygen-vacancy-related states closer to the conduction band, with the extracted TDOS evolution showing self-consistent correlation with the measured bias-stress-induced electrical behavior. Circuit-level validation with a complementary inverter confirms stable gain and noise margins. These results establish crystalline IGO as a viable single-material oxide channel combining high mobility, robust bias stability, and simplified processing for next-generation oxide-only backplanes.

## Graphical abstract

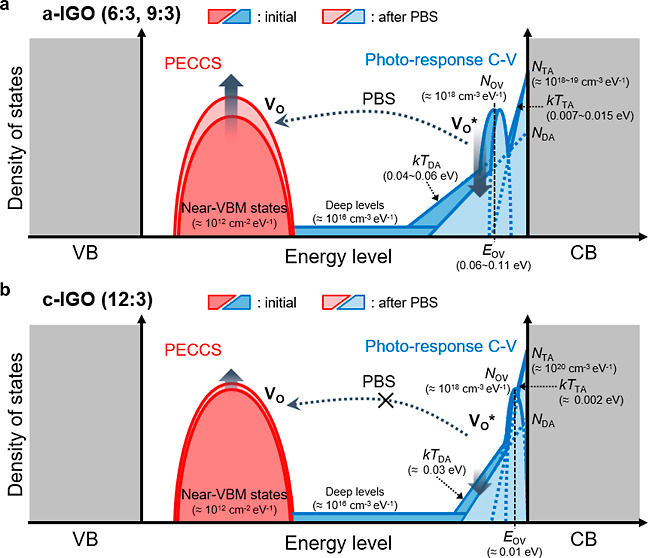

## Introduction

Since Hosono’s group reported amorphous indium–gallium–zinc oxide (a-IGZO) in 2004, oxide semiconductor thin-film transistors (TFTs) have attracted significant attention owing to their moderate mobility, low off-state leakage, large-area uniformity, and compatibility with low-temperature fabrication processes.^1–7^ In particular, a-IGZO offers these advantages in practice and has become the industry standard for TV-scale active matrix organic light-emitting diode (AMOLED) display backplanes.^8,9^ With the increasing demand for high-resolution and high-refresh-rate displays, the performance requirements have expanded to include a high drive-current capability, long-term stability under a high current density, and low leakage current. To address these requirements, low-temperature polycrystalline oxide (LTPO) architectures—integrating low-temperature polycrystalline silicon (LTPS) drive TFTs for high mobility with a-IGZO switching TFTs for low off-current—have been developed.^10,11^ This hybrid architecture has enabled ultrahigh resolution, fast response, and reduced power consumption in premium mobile OLED products. However, the increased process complexity of LTPO leads to higher manufacturing cost and a yield penalty.^12,13^

To reduce the process complexity, a single industry-compatible material that delivers high mobility comparable to LTPS while preserving the low leakage of amorphous oxide semiconductors is required. In this context, crystalline oxide semiconductors have the potential to satisfy these requirements, thereby enabling oxide-only backplanes as a feasible alternative to LTPO.^14–19^ However, conventional IGZO typically requires crystallization temperatures above 700 °C due to the Zn component, which restricts manufacturability for display applications.^20^ In this context, the Zn-free oxide semiconductor indium–gallium oxide (IGO) can mitigate this limitation: the absence of Zn enables low-temperature (≤400 °C) crystallization of high-quality oxide grains, often with preferential (222) orientation, thereby supporting higher mobility while maintaining low leakage.^14,17,21^

Implementing these recently developed oxide semiconductors for display backplanes requires not only high mobility and low leakage but also reliable operation that is directly governed by internal trap physics.^22^ For example, under positive-bias stress (PBS), donor-like oxygen vacancies in the doubly ionized state (V_O_^2+^) can capture two electrons and convert to neutral V_O_^0^, driving a positive shift in threshold voltage (*V*_TH_).^23,24^ In amorphous oxide semiconductors, oxygen-mediated metastable configurations, including peroxide models, have also been proposed under photo- or bias-stress conditions.^25^ In parallel, neutral-hydrogen-related processes near the channel–dielectric interface can also induce positive *V*_TH_ shifts.^26^ Moreover, as oxide TFTs increasingly adopt thin channel layers to enhance electrostatic control and suppress bulk-related disorder, carrier transport becomes confined closer to the channel–dielectric interface, making device performance more sensitive to the energetic distribution of trap states. Since such traps impact not only reliability but also core device metrics, rigorous energy-resolved trap analysis is essential. However, in oxide TFTs, trap characterization is still commonly performed by indirect methods, such as technology-computer-aided design (TCAD) simulation-based modeling or subthreshold swing (SS)-based estimates. These approaches cannot resolve the quantitative energy distribution of traps, which governs *V*_TH_ shift through shallow–deep transitions and mobility degradation through carrier trapping.^27–29^ In general, TCAD-based reproduction of transfer characteristics depends on multiple modeling choices including transport, contact, and trapping assumptions. Accordingly, such simulations often require careful calibration, as similar transfer characteristics can be reproduced even with different TDOS distributions depending on these modeling factors.^30^ In this light, a direct extraction of the trap density of states (TDOS), measured before and after bias stress and resolved near both the valence-band maximum (VBM) and the conduction-band minimum (CBM), has not been extensively explored for oxide TFTs.

In this work, we directly investigated the TDOS in ALD-grown amorphous and crystallized IGO TFTs using a combined optoelectronic characterization approach of photoexcited charge collection spectroscopy (PECCS) and photoresponse capacitance–voltage (C–V).^31–35^ With these complementary techniques, deep trap states near both the VBM and the CBM are quantified. Furthermore, the transition of trap states was monitored under both positive and negative bias stress (PBS and NBS) conditions. This experimental observation suggests that the high mobility and superior stability of the optimized IGO TFTs (In:Ga = 12:3) stem from V_O_-related states near the CBM (V_O_*) in crystalline IGO located closer to the CBM. By contrast, amorphous IGO is characterized by a larger contribution of deeper subgap states, which degrade carrier transport and bias stability. To validate the circuit-level feasibility of the crystalline IGO channel, we implemented a complementary inverter by integrating the n-channel IGO TFT with a p-Si transistor. These results confirm reliable operation at the circuit level and support the use of crystalline IGO for simplified oxide backplane technologies.

## Experimental Procedure

### Device Fabrication

Figure 1a shows a schematic of the fabricated IGO TFTs with bottom-gate and top-contact structures on a SiO_2_/Si substrate. The gate stack consists of 100 nm thick SiO_2_ as the gate insulator and a highly doped p-type Si substrate as the gate electrode. A 5 nm thick IGO thin film was first deposited on the SiO_2_ layer at 150 °C by a PEALD (Nexus BeCo. Ltd., South Korea). Dimethylbutylamino­(trimethylindium) (DATI) and trimethylgallium (TMG) (LakeMaterials Co., Ltd.) were used as In and Ga precursors, respectively, with argon (Ar) as the carrier gas at a flow rate of 50 sccm. The oxygen partial pressure (PO_2_) was maintained at 50%, and the plasma power was set to 150 W. The IGO thin films with three different In:Ga compositions (6:3, 9:3, and 12:3) were deposited by adjusting the ratio of binary metal oxide subcycles. The deposited films were patterned by using conventional photolithography and wet etching techniques. Subsequently, a 100 nm thick indium tin oxide (ITO, In/Sn = 9:1 wt %) film was deposited by DC magnetron sputtering.^36^ The source/drain electrodes were defined by photolithography and lift-off processes, with a channel width (*W*) and length (*L*) of 60 and 30 μm, respectively. Postdeposition annealing was conducted in air at 400 °C for 1 h. Finally, a 5 nm thick gallium oxide (Ga_2_O_3_) passivation layer was deposited by PEALD at 250 °C. The number of batches fabricated for each composition was 2, with 24 devices in each batch.

**Figure 1:**
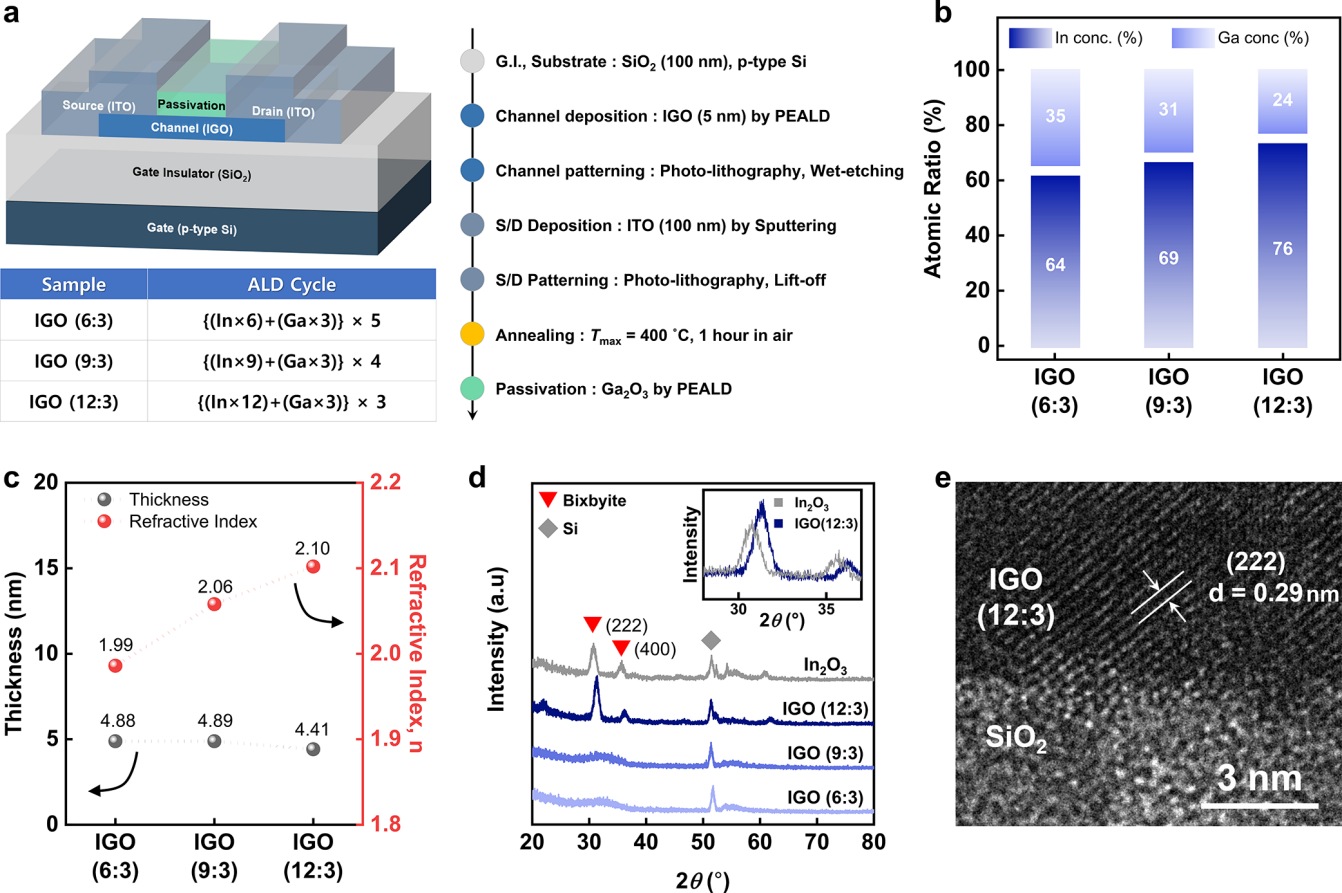
a) Structure of the fabricated TFTs and fabrication process, with the ALD supercycle duty used for channel deposition for each sample. (b) Cation composition ratios extracted by XRF analysis, (c) film thickness and refractive index, and (d) XRD results of each annealed In_2_O_3_ and IGO films. (e) TEM image of crystalline IGO TFTs.

### Electrical Measurements

Electrical characteristics were measured in the dark using an HP 4155B semiconductor parameter analyzer. Capacitance–voltage (C–V) was measured with an HP 4284A precision LCR meter. All measurements were conducted at room temperature (23 ± 2 °C), and a relative humidity was maintained at 30%–40% to minimize the effects of moisture on the device’s performance. The field-effect mobility (μ_FE_) was extracted in the linear regime at a low drain bias (*V*_DS_ = 0.1 V) using μ_FE_ = *L* × *g*_m_/(*W* × *C*_OX_ × *V*_DS_), where *g*_m_ is the transconductance, *C*_OX_ is the gate capacitance per unit area, and *V*_DS_ is the drain-source voltage. The threshold voltage (*V*_TH_) was determined by the constant-current method, defined as the gate voltage (*V*_GS_) corresponding to a drain current (*I*_DS_) of *L*/*W* × (10 nA), and the subthreshold swing (SS) was extracted using SS = d*V*_GS_/d log­(*I*_DS_), both from transfer characteristics measured under saturation conditions at *V*_DS_ = 5.1 V.

## Results and Discussion

### Material Characterization

IGO channel layers with three distinct In:Ga cation fractions of 6:3, 9:3, and 12:3 were deposited by plasma-enhanced atomic layer deposition (PEALD) by adjusting the subcycle duty during processing. The schematic structure of the IGO TFT and the corresponding PEALD fabrication process with specific duty settings are summarized in Figure 1a. An optical microscopy image of the fabricated TFT is presented in Figure S1. Figure 1b shows the atomic ratios extracted from X-ray fluorescence (XRF) measurements under each process condition. Consistent with the ideal mixture rule for the In–O/Ga–O binary system, the growth per cycle of the In–O and Ga–O subcycles remained constant across different subcycle duties, allowing deterministic control of both stoichiometry and thickness.^14^ The thickness of the IGO channel was kept nearly constant to isolate composition-dependent electrical properties, while the refractive index increased with the indium ratio, as measured by ellipsometry (Figure 1c). The crystalline phase of the IGO channel was determined by the chemical composition. As shown in Figure 1d, the grazing incidence X-ray diffraction (GI-XRD) patterns of IGO (6:3) and IGO (9:3) after 400 °C postdeposition annealing showed no notable diffraction peaks, consistent with an amorphous phase. In contrast, IGO (12:3) exhibited a diffraction peak corresponding to crystalline In_2_O_3_ with a cubic bixbyite structure. While the bixbyite crystal framework is retained, the diffraction peak is shifted relative to that of pristine In_2_O_3_, reflecting lattice-parameter modification induced by Ga incorporation. Importantly, the shifted peak position matches reported bixbyite In_1.6_Ga_0.4_O_3_ (IGO (12:3)) reference patterns rather than those of pure In_2_O_3_. This confirms that crystalline IGO preserves the bixbyite structure while exhibiting a smaller lattice spacing compared to In_2_O_3_, originating from Ga substitution.^14^ The formation of the bixbyite microstructure with a preferential (222) orientation was further confirmed by cross-sectional high-resolution transmission electron microscopy (HR-TEM) for the 12:3 film (Figure 1e).

### Device Characterization

Figures 2a–c present transfer characteristics of IGO TFTs with different compositions, all of which exhibit negligible hysteresis and low off-current of ≈10^–13^ A. The low off-current results from the wide band gap that suppresses thermally generated carriers and the unipolar n-type conduction that prevents hole inversion under reverse bias. The corresponding device performance parameters are summarized in Figure 2d and Table 1. Here, a total of 20 devices were measured, with 10 devices randomly selected from each of the 2 batches. No significant differences were observed among the batches. As the Ga fraction decreased, the field-effect mobility increased from 26.1 to 83.3 cm^2^ V^–1^ s^–1^, while the threshold voltage shifted from 1.69 to 0.33 V. This trend arises because the bond energy of Ga–O (374 kJ mol^–1^) exceeds that of In–O (346 kJ mol^–1^). The stronger Ga–O bonds suppress oxygen-vacancy formation and thus free-carrier density; therefore, reducing the Ga content relaxes this suppression and enhances carrier transport.^17,37,38^ At an In:Ga ratio of 12:3, the mobility increased sharply to 83.3 ± 1.3 cm^2^ V^–1^ s^–1^, which is significantly higher than that of devices with other compositions. This abrupt mobility enhancement at a 12:3 composition arises from a composition-induced amorphous-to-crystalline transition, which facilitates low-scattering-rate transport pathways. A literature-based discussion on the role of oxygen-vacancy-related trap states in governing device performance metrics, along with representative control strategies, is provided in Supporting Information Note S1. Furthermore, no discernible changes in the transfer characteristics were observed for both amorphous and crystalline devices over an observation period of up to 58 days (Figure S2).

**Figure 2:**
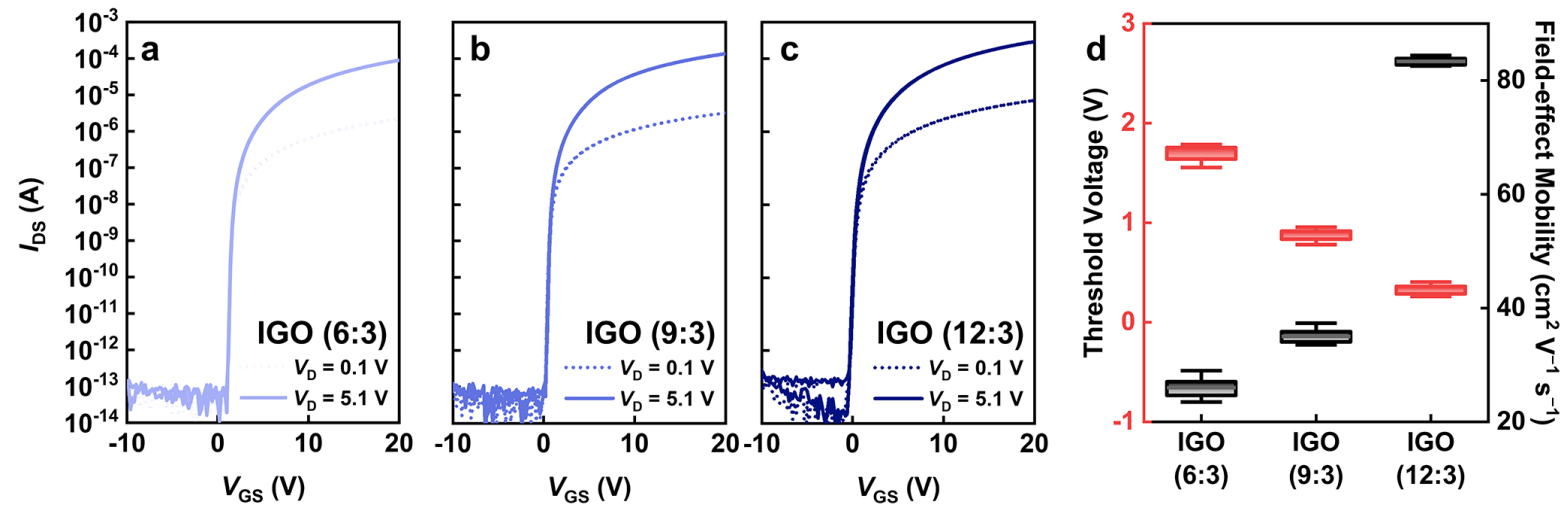
Transfer characteristics of IGO TFTs with compositions of (a) 6:3, (b) 9:3, and (c) 12:3. (d) Box plots of *V*_TH_ and mobility extracted from 20 devices under each condition.

**Table 1:** Extracted Device Parameters of IGO TFTs with Varying Cation Composition, Obtained from 20 Devices under Each Condition

sample	IGO (6:3)	IGO (9:3)	IGO (12:3)
μ_FE_ (cm V^–1^ s^–1^)	26.0 ± 1.7	35.0 ± 1.2	83.4 ± 0.62
SS (V dec^–1^)	0.09 ± 0.04	0.11 ± 0.02	0.14 ± 0.03
*V*_TH_ (V)	1.69 ± 0.07	0.88 ± 0.05	0.33 ± 0.05
*I*_ON/OFF_	>10^9^	>10^9^	>10^9^

The bias stability of the devices was further evaluated by applying gate-bias stress under gate-to-source voltage (*V*_GS_) at *V*_TH_ ± 20 V for 3600 s, corresponding to a calculated *E*_stress_ = (*V*_GS,stress_ – *V*_TH_)/*t*_OX_ of approximately ±2 MV cm^–1^ for an oxide thickness (*t*_OX_) of 100 nm. After PBS, the Δ*V*_TH_ of the Ga-rich 6:3 device reached +1.2 V, whereas the In-rich 12:3 device showed only +0.44 V (Figure 3a–c). Under NBS, all devices exhibited Δ*V*_TH_ <0.1 V (Figure 3d–f). The reproducibility of the PBS stability behavior was confirmed across three devices, showing minimal variation (Figure S3). Overall, the In-rich crystalline IGO (12:3) exhibited the best stability (Δ*V*_TH_ <0.5 V), clearly outperforming the Ga-rich amorphous devices (Figure S4).

**Figure 3:**
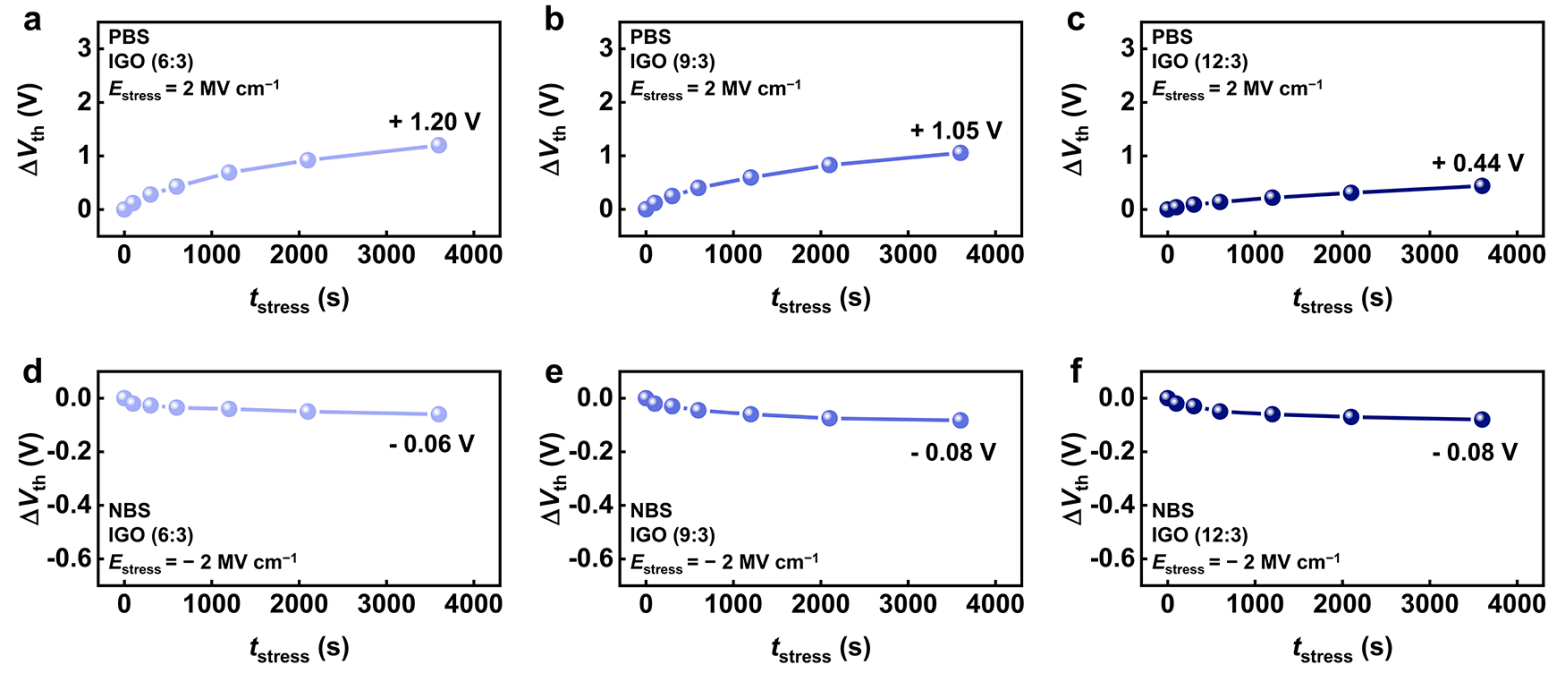
*V*_TH_ shifts of IGO TFTs with compositions of (a–c) 6:3, 9:3, and 12:3 under positive bias stress and (d–f) under negative bias stress.

### TDOS Characterization using PECCS

To clarify the origin of bias stability, TDOS within the band gap was extracted and correlated with the observed electrical behavior under bias stress. Specifically, the TDOS near the VBM and CBM was characterized using complementary optoelectronic measurements based on PECCS and photoresponse C–V, respectively, both before and after bias stress to track the evolution of the trap-state distribution. Figure 4a and b outline the principle of PECCS.^31,39^ In this measurement, when the light with a photon energy (*h*ν) smaller than the optical band gap is applied, charges trapped in gap states located at energies up to *h*ν below the CBM are released by incident photons and collected at the source and drain electrodes, thereby inducing a shift in *V*_TH_. Then, the trap density (*D*_it_) can be extracted from the spectral derivative of the threshold shift as

1 $$D_{it}\;\left(E_{CBM}-h\nu \right)=\left(\frac{C_{OX}}{q}\right)\;\frac{dV_{TH\;}\left(h\nu \right)}{dh\nu }$$

where *E*_CBM_ is the conduction band minimum energy, *h*ν is the incident photon energy, and *q* is the elementary charge. A Xe lamp and a monochromator provided excitation from 700 to 400 nm in 5 nm steps. This photon-energy range lies entirely within the optical band gap (∼3.6 eV) of the IGO devices, ensuring sub-band gap excitation during PECCS measurements (Figure S5). To avoid artifacts from a drain bias (*V*_DS_), the measurements were performed in the linear regime with a small *V*_DS_ of 0.1 V. Because all trap states must be occupied prior to photoexcitation, each sweep was initiated from the accumulation state, with *V*_GS_ swept from +10 to −10 V for every acquisition. Figure 4c presents the pristine TDOS of the amorphous and crystalline IGO TFTs. Both devices exhibit near-VBM states broadly distributed up to ≈1.5 eV above the VBM, with a peak near ≈0.7 eV corresponding to the V_O_^0^ state.^40^ As the Ga ratio decreases, the V_O_^0^ density increases because Ga suppresses V_O_^0^ formation by stabilizing oxygen bonds.^3^ After PBS, the TDOS distribution exhibited noticeable changes with the near-VBM states of the three devices showing distinct behaviors, as shown in Figure 4d–f. The amorphous 6:3 and 9:3 devices showed a significant increase in near-VBM states. In contrast, the crystalline 12:3 device exhibited the least change, despite containing the highest V_O_^0^ density. The time-dependent evolution of the TDOS in IGO TFTs is examined in Figure S6, showing a continuous transition without noticeable generation of additional peaks. On the other hand, after NBS, all three samples remained stable, with no noticeable change in the extracted TDOS (Figure 4g–i).

**Figure 4:**
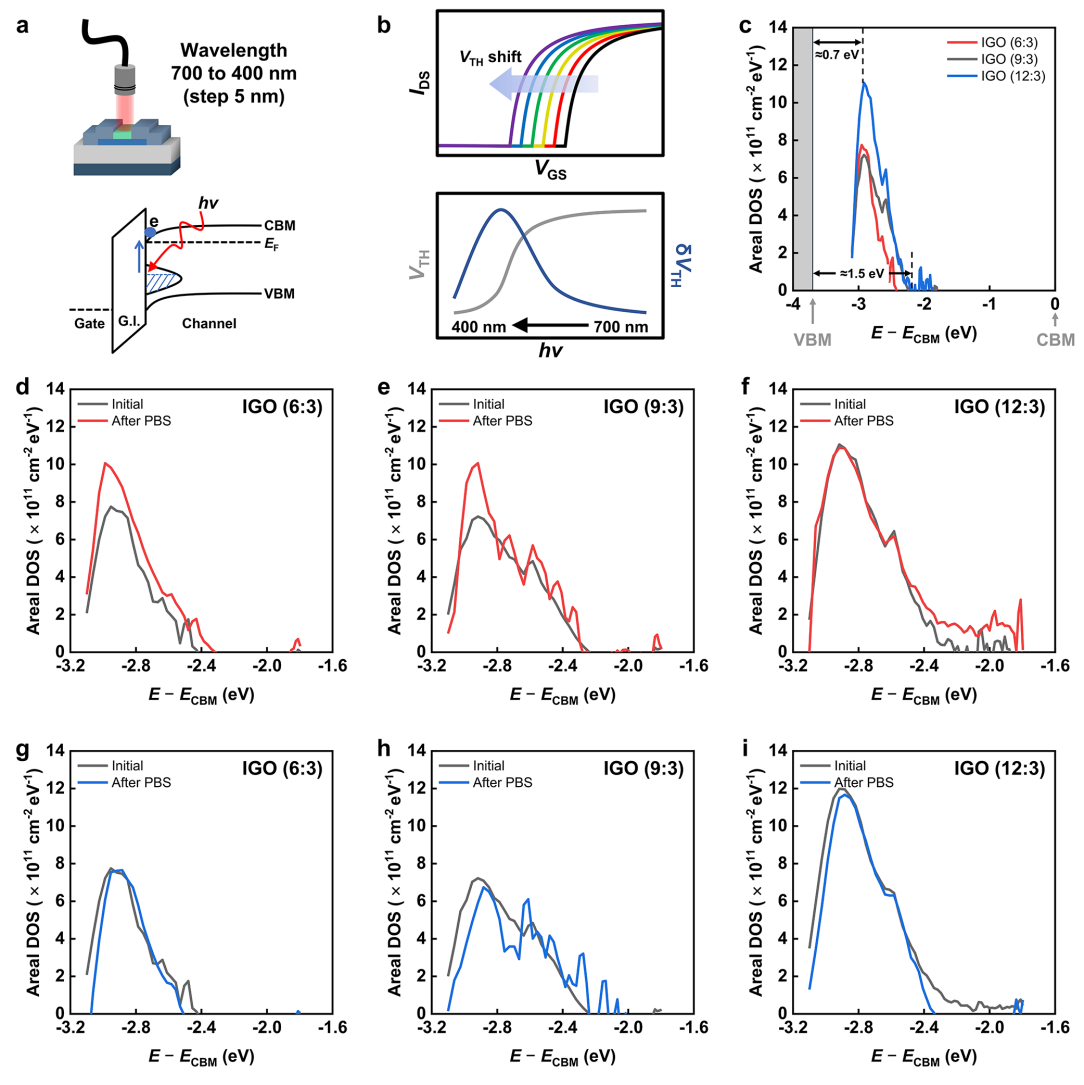
a,b) Schematic illustration and measurement principle of the PECCS method for extracting near-VBM states within the band gap. (c) Initial TDOS of IGO TFTs with compositions of 6:3, 9:3, and 12:3. TDOS changes of the corresponding devices after (d–f) PBS and (g–i) NBS.

### TDOS Characterization using Photoresponse C–V

Next, the acceptor-like TDOS near the CBM was quantified using photoresponse C–V, and the measurement principle is depicted in Figure 5a.^33,34^ During C–V under monochromatic illumination, electrons are photoexcited from localized states within the energy range (*E*_CBM_ – *h*ν) < *E* < *E*_F_, where *h*ν is the incident photon energy and *E*_F_ is the Fermi level. The released charge (Δ*Q*) introduces an additional parallel capacitance in the channel (*C*_ph_), thereby increasing the measured total capacitance. As shown in Figure 5b, the total measured capacitance (*C*_m_) consists of *V*_GS_-independent components such as source/drain overlap and parasitic capacitance (*C*_OV_ and *C*_par_) and *V*_GS_-dependent components (*C*_m,dark_ or *C*_m,ph_) originating from the channel. The parasitic capacitance (*C*_par_) was evaluated by multiplying the gate-oxide capacitance density measured from channel-free test structures by the parasitic capacitance area, defined as the nonchannel gate–source/drain overlap area extracted from the actual device geometry. By comparing the *V*_GS_-dependent components of the dark and illuminated C–V traces, the number of carriers released from traps can be extracted and subsequently converted to the trap density of states (*g*(*E*)) using the following equations,

2 $$\frac{1}{C_{m,dark}\left(V_{GS}\right)}=\frac{1}{C_{OX}WL}+\frac{1}{C_{CH}\left(V_{GS}\right)}$$

3 $$\frac{1}{C_{m,ph}\left(V_{GS}\right)}=\frac{1}{C_{OX}WL}+\frac{1}{C_{CH}\left(V_{GS}\right)+C_{ph}\left(V_{GS}\right)}$$

4 $$g\left(E\right)=\frac{dN_{T}}{dE}=\frac{dN_{T}}{qdV}=\frac{1}{q^{2}WLt_{CH}}\;\frac{dQ}{dV}=\frac{C_{ph}\left(V_{GS}\right)}{q^{2}WLt_{CH}}\;\left[J^{-1}{cm}^{-3}\right]=\frac{C_{ph}\left(V_{GS}\right)}{qWLt_{CH}}\;\left[{eV}^{-1}{cm}^{-3}\right]$$

where *C*_m,dark_ and *C*_m,ph_ denote the measured capacitances under dark and illumination conditions, respectively. *C*_CH_ is the channel capacitance under a dark condition, and *C*_OX_ is the gate-oxide capacitance per unit area. *N*_T_ and *V* are the number of trap states and channel potential, respectively. *t*_CH_, *W*, and *L* are the channel thickness, width, and length, respectively. The *C*_m,dark_ and *C*_m,ph_ obtained from 6:3, 9:3, and 12:3 IGO TFTs are represented in Figure S7.^41^ The effective channel length (*L*_eff_), defined by the following equation, was used in place of *L* in eq 4 to account for the gate-voltage-dependent variation of the electrically active channel.^42^

5 $$L_{eff}\;\left(V_{GS}\right)=L\;\frac{C_{CH}\left(V_{GS}\right)}{C_{OX}WL-C_{OV}}$$

**Figure 5:**
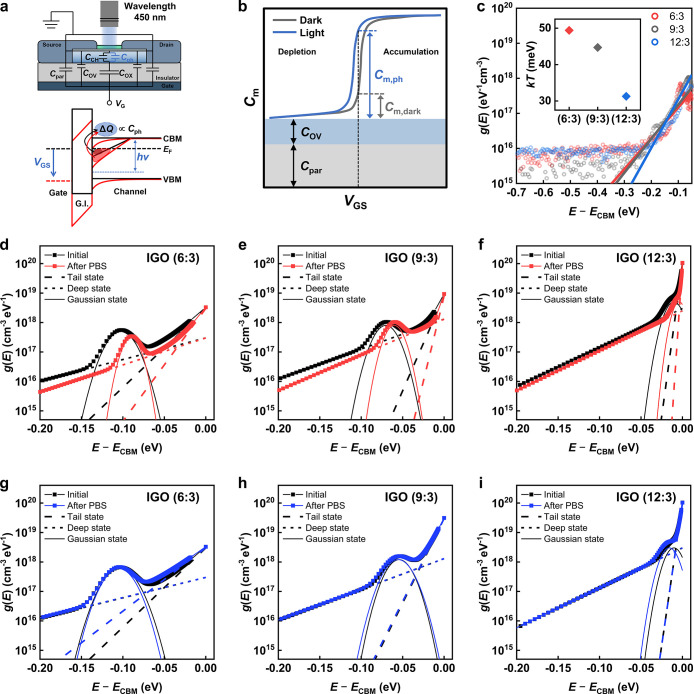
a) Schematic illustration and (b) measurement principle of the photoresponse C–V method for extracting near-CBM acceptor-like states. (c) Initial TDOS of IGO TFTs with compositions of 6:3, 9:3, and 12:3. The inset shows the extracted characteristic energy width, corresponding to the inverse of the exponential slope. TDOS changes of the corresponding devices after (d–f) PBS and (g–i) NBS.

Finally, the surface potential (ψ_S_) was extracted from the measured C–V via the following equation.^41^

6 $$q\psi_{S}=E_{CBM}-E=q\int_{V_{FB}}^{V_{GS}}\;\left(1-\frac{C_{m,dark}}{C_{OX}WL}\right)\;dV_{GS}$$

Consequently, the extracted *g*(*E*) as a function of *E* – *E*_CBM_ can be obtained using *V*_GS_ as an intermediate parameter through eqs 4 and 6. During the measurement, the wavelength of illumination was fixed at 450 nm, corresponding to a photon energy of ≈2.76 eV, ensuring sub-band gap excitation and selective probing of acceptor-like trap states near the CBM without inducing band-to-band transitions (Figure S5). During the measurement, the wavelength of light was fixed at 450 nm and the voltage sweep was initiated from the accumulation regime. The extracted acceptor-like TDOS is depicted in Figure 5c. The characteristic energy width for crystalline IGO (12:3) decreased to 31.3 meV, compared to 44.7 meV for the amorphous device (9:3), and 49.4 meV for the amorphous device (6:3), confirming a significant steepening of the trap distribution near the conduction band edge. A steeper slope of acceptor-like traps generally correlates with the improved mobility and bias stability.^28,43,44^ To further clarify the origin of this trend and resolve the detailed near-CBM structure, TDOS was fitted with two exponential components and one Gaussian component to identify the specific trap components contributing to this correlation.

7 $$g\left(E\right)=g_{TA}\left(E\right)+g_{DA}\left(E\right)+g_{OV}\left(E\right)$$

8 $$g_{TA}\left(E\right)=N_{TA}\;\exp\left(\frac{E-E_{CBM}}{kT_{TA}}\right)$$

9 $$g_{DA}\left(E\right)=N_{DA}\;\exp\left(\frac{E-E_{CBM}}{kT_{DA}}\right)$$

10 $$g_{OV}\left(E\right)=N_{OV}\;\exp\left(\frac{{-\left(\left(E-E_{CBM}\right)-E_{OV}\right)}^{2}}{{\left(kT_{OV}\right)}^{2}}\right)$$

Here, *g*_TA_(*E*) represents the acceptor-like tail state, *g*_DA_(*E*) represents the acceptor-like deep state, and *g*_OV_(*E*) represents the Gaussian state. And *N*_TA_/*N*_DA_ denote the prefactor of the acceptor-like tail/deep state, *kT*_TA_/*kT*_DA_ are the characteristic energy width of the tail/deep exponential states, *N*_OV_ is the peak amplitude of the Gaussian state, *E*_OV_ is the peak position relative to CBM, and *kT*_OV_ is the Gaussian width parameter. The measured data and extracted model parameters are shown in Figure S8 and Tables S1 and S2.

Figure 5d–i shows that with increasing In fraction, the Gaussian states shift closer to the CBM, while the slopes of the tail and deep exponential states become steeper. After PBS, all three devices exhibited a decrease in the width of the exponential states (i.e., a steeper slope) and a reduction in the magnitude of the Gaussian states, where the 6:3 and 9:3 amorphous devices showed notable decreases, while the 12:3 crystalline device showed only minimal changes (Figure 5d–f). In particular, the characteristic energy widths of deep exponential states decreased more than those of the tail states (Figure S9). As shown in Figure S10, the TDOS of IGO TFTs evolves smoothly over time with no evident emergence of new peaks. In contrast, NBS induced a negligible change in the acceptor-like TDOS for any of the three devices (Figure 5g–i). The detailed experimental parameters and reproducibility controls for the PECCS and photoresponse C–V measurements, including light-intensity calibration, gate-voltage sweep direction and rate, AC excitation conditions, and measurement frequency, are provided in Supporting Information Note S2.

### Comprehensive Analysis of TDOS

By correlating complementary measurements of near-VBM trap states from PECCS and near-CBM trap states from photoresponse C–V, we comprehensively tracked the bias-induced evolution of the total TDOS before and after bias stress in amorphous and crystalline IGO TFTs. This enables energy-resolved tracking of stress-induced trap redistribution that has been discussed in terms of oxygen-vacancy charge-state transitions and oxygen-mediated metastable configurations. In the amorphous devices (6:3, 9:3), the V_O_* states near the CBM are located farther from the CBM, which leads to a decrease in V_O_* states and a corresponding increase in V_O_^0^ states near VBM. In addition, the exponential distribution of deep traps near the CBM becomes shallower after PBS, indicating that a portion of these deep traps is also reduced or redistributed toward the low-energy region within the band gap (Figure 6a). In contrast, the V_O_* states are positioned closest to the CBM in the crystalline device (12:3), and both the Gaussian center and the exponential deep-trap distribution remain nearly unchanged after PBS (Figure 6b). Notably, no qualitatively distinct defect-related peaks are observed in crystalline IGO compared with the amorphous devices, suggesting that the oxygen-vacancy-related photoinstability mechanism established for amorphous oxide semiconductors can be extended to crystalline oxide semiconductors.^23–26^ Consequently, the comparative TDOS analysis demonstrates that in crystalline IGO, V_O_* states are confined near the CBM and exhibit the smallest stress-induced variation, whereas in amorphous IGO, V_O_* states are located deeper in the band gap and undergo more pronounced evolution under bias stress. This TDOS behavior is fully consistent with the observed PBS stability characteristics, indicating from direct experimental evidence that the superior bias stability of crystalline IGO TFTs arises from the favorable energetic alignment of V_O_* states near the CBM.^22,45–47^ Moreover, these states act as shallow donor-like states that enhance conductance and carrier mobility (Figure S11). Consequently, these results confirm that the energetic alignment of trap states relative to the CBM is the key factor governing both bias stability and mobility, with crystalline ordering providing the best energetic alignment for simultaneous improvements in reliability and carrier transport.^45,48^ The methodological scope and limitations of the optoelectronic characterization employed in this study, including a comparison with multifrequency C–V and model-based TDOS reconstruction methods and clarification of the effective TDOS interpretation under photobias conditions, are discussed in Supporting Information Note S3.

**Figure 6:**
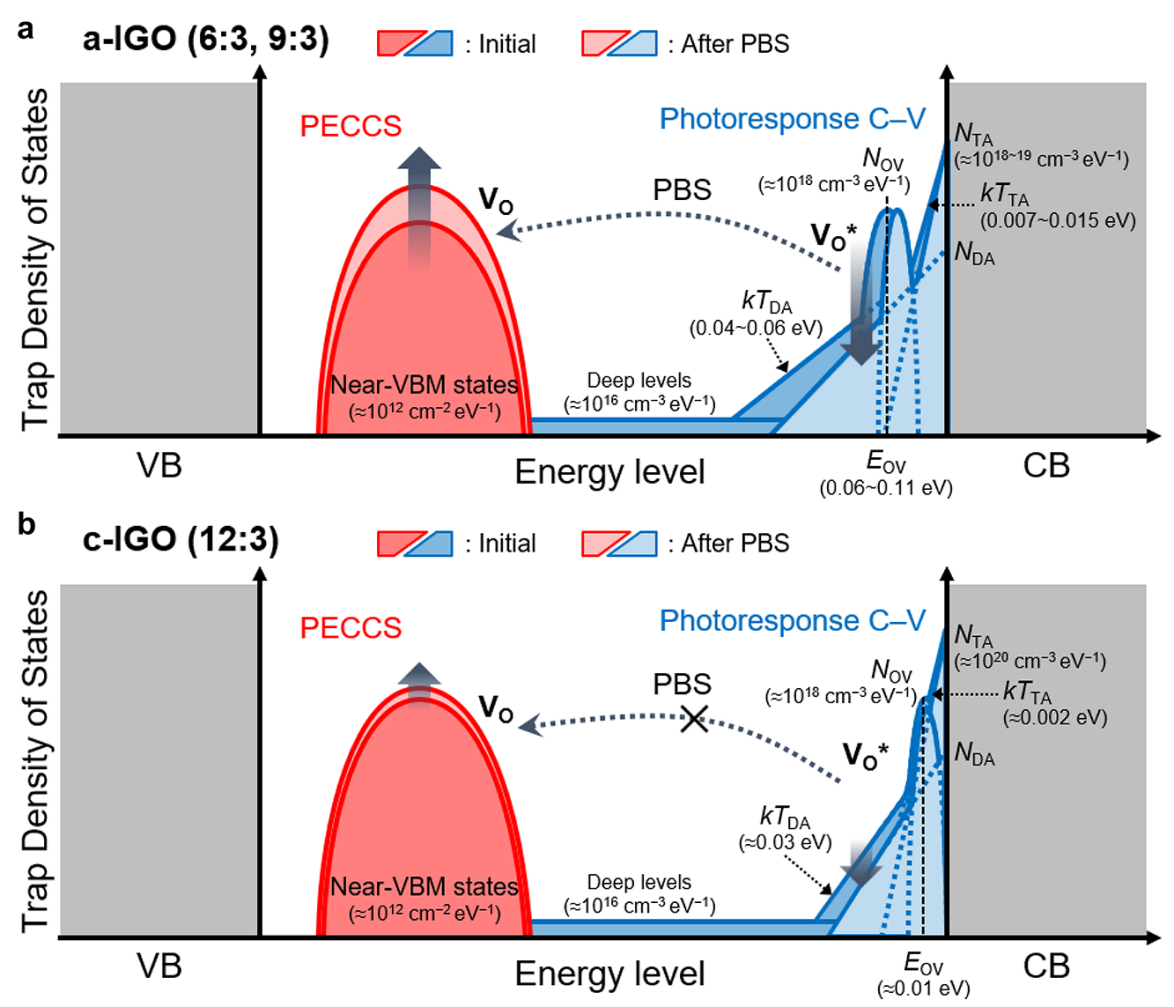
Schematic illustration of the subgap TDOS of (a) amorphous IGO TFTs and (b) crystalline IGO TFTs. The near-VBM states were extracted using PECCS, and the near-CBM acceptor-like states were extracted using photoresponse C–V.

### Implementation of a Complementary Inverter

To validate the circuit-level performance of the crystalline IGO TFTs, we implemented a complementary inverter by pairing the optimized n-channel IGO TFT (In:Ga = 12:3) with a p-Si transistor. Figure 7a shows the circuit configuration of the inverter. The voltage transfer characteristics measured at supply voltages from 1 to 5 V are presented in Figure 7b, showing distinct logic inversion over the entire input range. The corresponding voltage gain, extracted from the slope of the transfer curves, increased with supply voltage and reached a value of 647 at *V*_DD_ = 5 V (Figure 7c). Both noise margins scaled monotonically with the supply voltage (Figure 7d). Endurance was further examined through repeated switching measurements over 520 consecutive sweeps. As shown in Figure 7e, the output characteristics retained their sharp transition without noticeable degradation (<0.05 V). Statistical analysis revealed that both the gain (Figure 7f) and the noise margins (Figure 7g) exhibited only minimal variations over 520 cycles and no degradation was observed under repeated operation. These results confirm that the crystalline IGO TFTs support circuit-level operation, sustaining high gain and robust noise margins over extended cycling. Our key device performance metrics of optimized crystalline IGO TFTs are summarized in Table S3. Beyond the verified device-level stability, the circuit-level validation highlights the potential of crystalline IGO as a high-performance and reliable n-type channel for simplified oxide backplanes.

**Figure 7:**
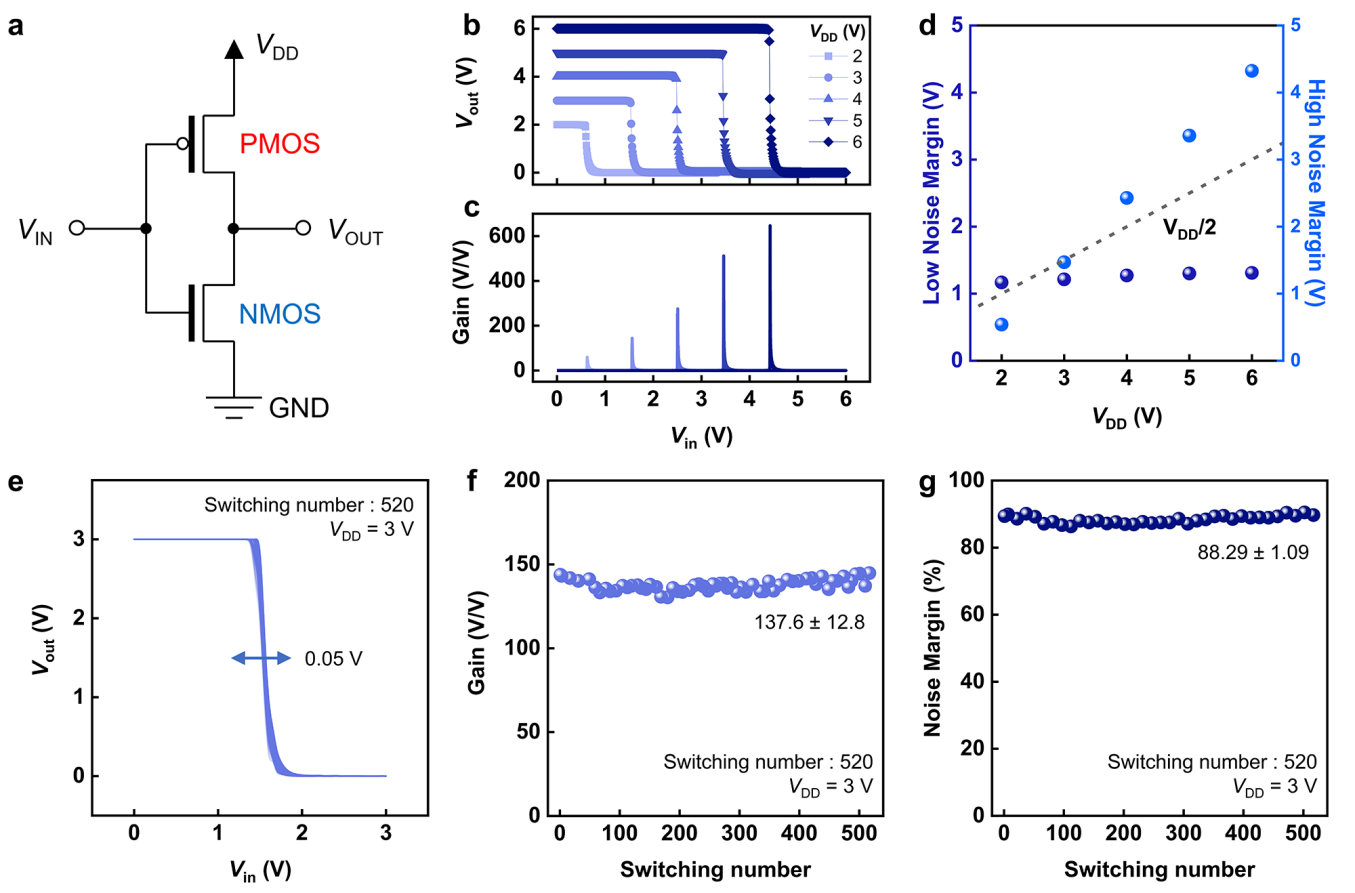
a) Circuit diagram of the complementary inverter. (b) Voltage transfer characteristics. (c) Voltage gain extracted from the transfer curves as a function of *V*_DD_. (d) Low and high noise margins as a function of *V*_DD_. (e) Voltage transfer characteristics measured over 520 consecutive sweeps, confirming stable operation. (f) Distribution of voltage gain extracted from 520 consecutive sweeps. (g) Distribution of noise margins extracted from 520 consecutive sweeps.

## Conclusion

By controlling cation stoichiometry in Zn-free indium–gallium oxide, we induced a composition-driven amorphous-to-crystalline transition at an annealing temperature below 400 °C, enabling thin-film transistors with mobility up to 83.3 cm^2^ V^–1^ s^–1^ and an off-current as low as ≈10^–13^ A. Statistical analysis across 20 devices fabricated from two independent batches confirmed highly reproducible electrical characteristics with negligible batch-to-batch variation. Bias stress stability was verified through PBS and NBS measurements, in which the optimized crystalline IGO TFTs (In:Ga = 12:3) exhibited a threshold-voltage shift of less than 0.5 V. The trap density of states before and after bias stress was quantitatively analyzed using complementary PECCS and photoresponse C–V measurements. Taken together, the extracted TDOS and the measured bias-stability trends provide experimental evidence that, in crystalline IGO TFTs, oxygen-vacancy-related states are located closer to the CBM, and this shallower V_O_* state distribution is associated with smaller bias-stress-induced changes. In contrast, in amorphous IGO, the corresponding states are located deeper in the band gap and undergo a more pronounced stress-induced evolution, leading to bias instability. Circuit-level validation with a complementary inverter further confirmed stable gain and noise margins under repeated switching, highlighting the reliability of crystalline IGO TFTs in practical operation. These results establish crystalline IGO as a promising single-material oxide channel for oxide-only AMOLED backplanes, offering simplified processing with performance comparable to that of LTPO technologies.

## Supplementary Material



## Notes

Supplementary figures and tables detailing device images, bias-stress characteristics, MPCV-derived TDOS analysis, and extracted trap parameters for amorphous and crystalline IGO TFTs (PDF)
