# Supplementary material for: Optoelectronic Characterization of Trap Density of States in Indium Gallium Oxide Thin-Film Transistors and Their Impact on Bias Stability
Source: ACS Appl Mater Interfaces. 2026 Feb 19;18(8):13100–10. doi: 10.1021/acsami.5c21764 (PMC12964345; doi:10.1021/acsami.5c21764)
Supplement: Supplementary file 1 [file am5c21764_si_001.pdf]

Supporting Information

Optoelectronic Characterization of Trap Density of States in Indium Gallium Oxide Thin-Film Transistors and Their Impact on Bias Stability

Sang Yeon Kim[1,2,†], Je-Jun Lee,[1,†], Jae Seok Hur[2], Buyeon Kim[2], Jung Pyo Hong[1,3], Seong-Jun Han[1,3], Eungseon Yeon[1,3], Jung Woo Kim[1,3], Jae Kyeong Jeong[2,*], and Do Kyung Hwang[1,3,4,*]

[1] Center of Quantum Technology, Post-Silicon Semiconductor Institute, Korea Institute of Science and Technology (KIST), Seoul 02792, Republic of Korea.

[2] Department of Electronic Engineering, Hanyang University, Seoul 04763, Republic of Korea.

[3] KU-KIST Graduate School of Converging Science and Technology, Korea University, Seoul 02841, Republic of Korea.

[4] Division of Nanoscience & Technology, KIST School, University of Science and Technology (UST), Seoul 02792, Republic of Korea.

* Corresponding Authors' E-mail: jkjeong1@hanyang.ac.kr (J. K. Jeong) and dkhwang@kist.re.kr (D. K. Hwang)

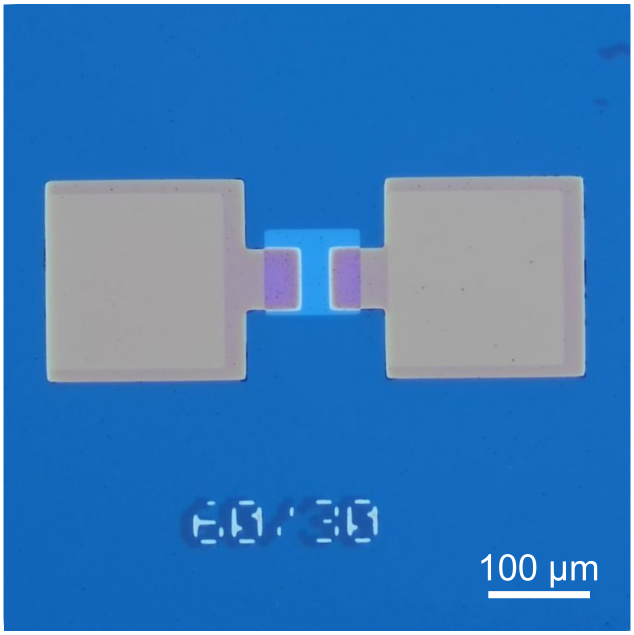

Figure S1. Optical microscopy image of IGO TFT. Scale bar is 100 μm.

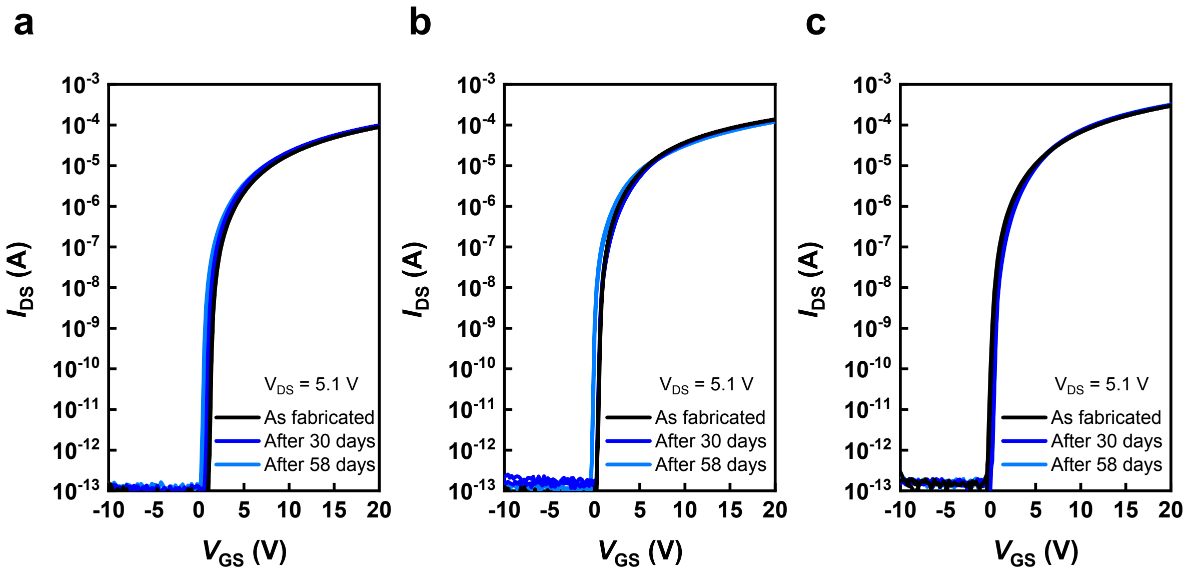

Figure S2. Transfer characteristics of (a) IGO (6:3), (b) IGO (9:3), and (c) IGO (12:3) devices measured in ambient air at room temperature for up to 58 days.

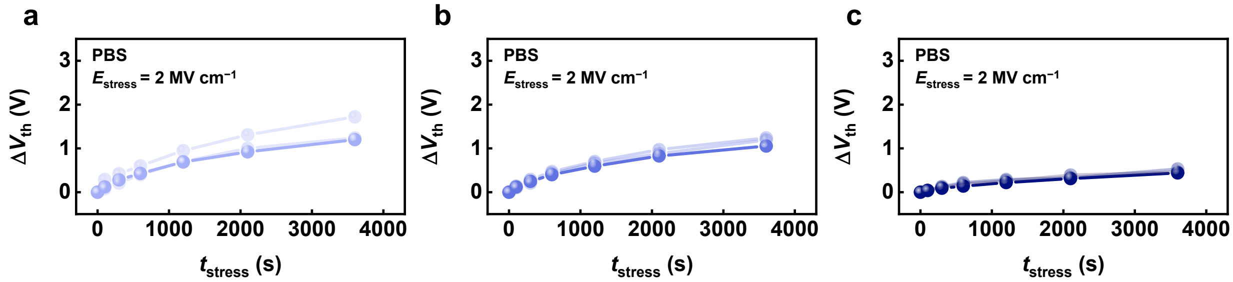

Figure S3. Reproducibility of PBS stability measured across three individual IGO TFTs for (a) IGO (6:3), (b) IGO (9:3), and (c) IGO (12:3).

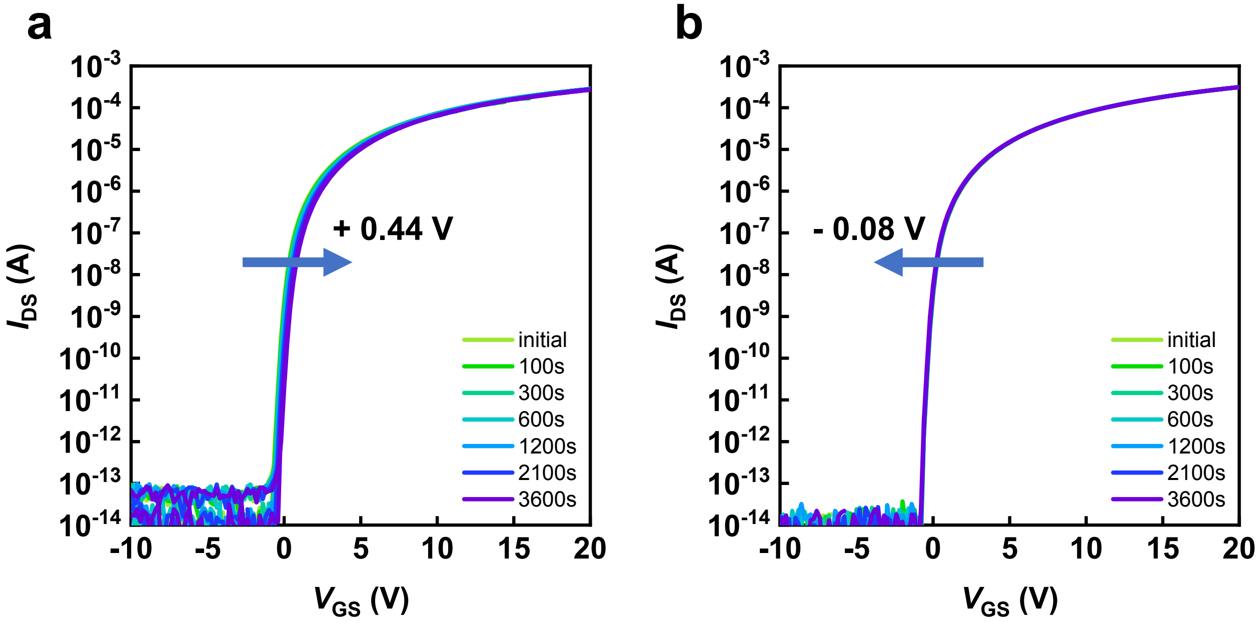

Figure S4. Transfer characteristics of crystalline IGO TFT under (a) PBS and (b) NBS condition.

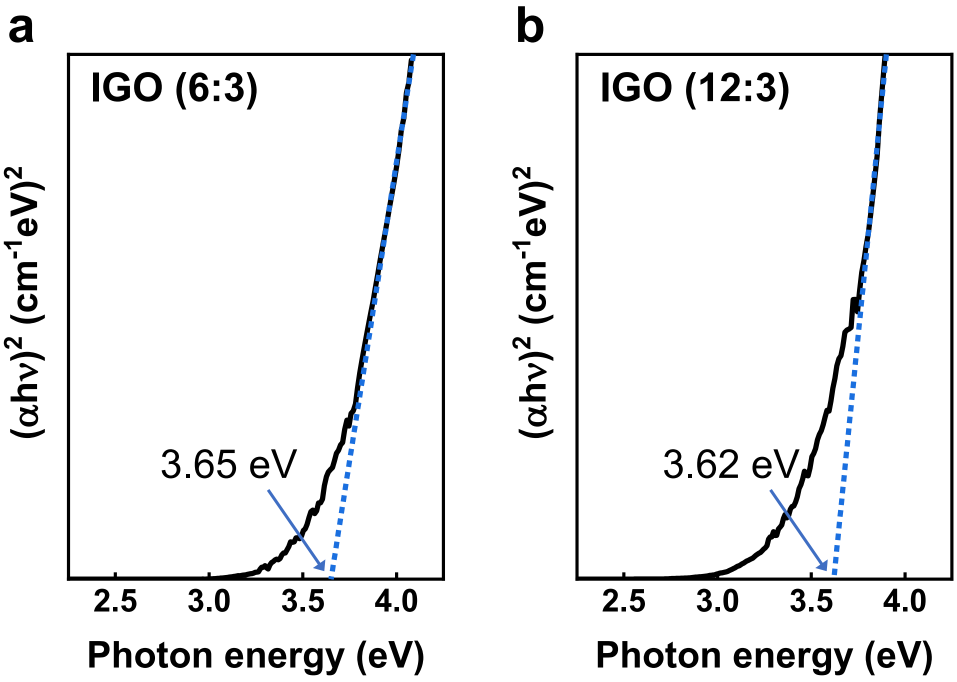

Figure S5. Tauc plots derived from UV–vis spectra of (a) IGO (6:3) and (b) IGO (12:3).

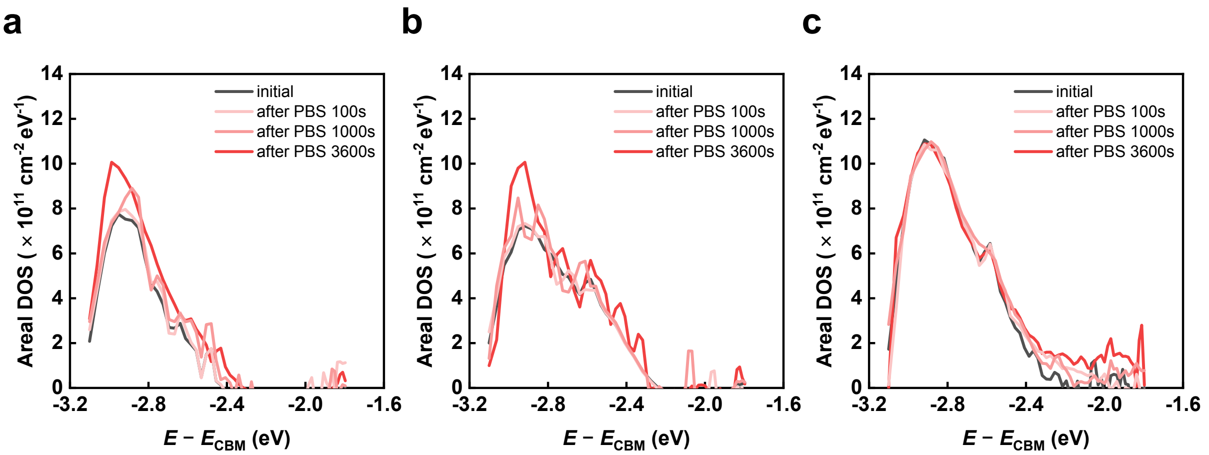

Figure S6. Time-dependent TDOS of IGO TFTs with compositions of (a) 6:3, (b) 9:3, and (c) 12:3 under positive bias stress, obtained using PECCS.

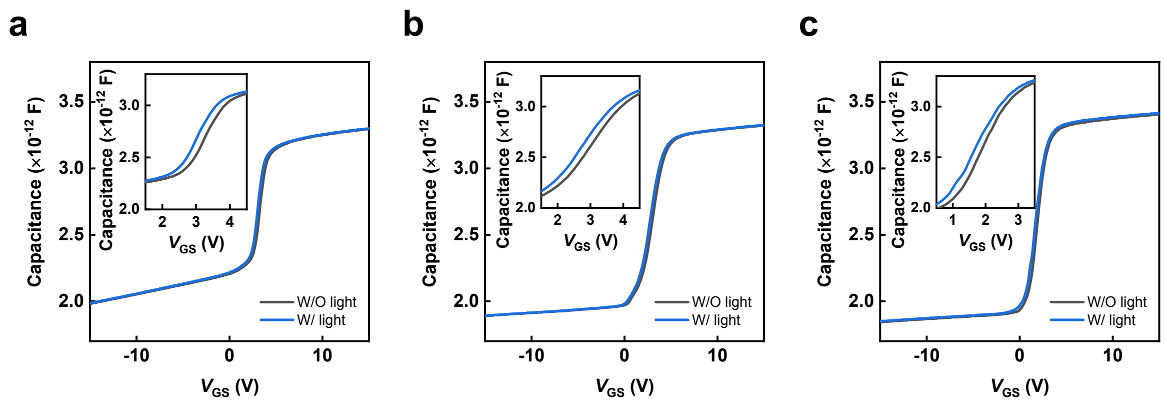

Figure S7. Measured capacitance-voltage characteristics under dark and illuminated conditions for (a) IGO (6:3), (b) IGO (9:3), and (c) IGO (12:3) devices.

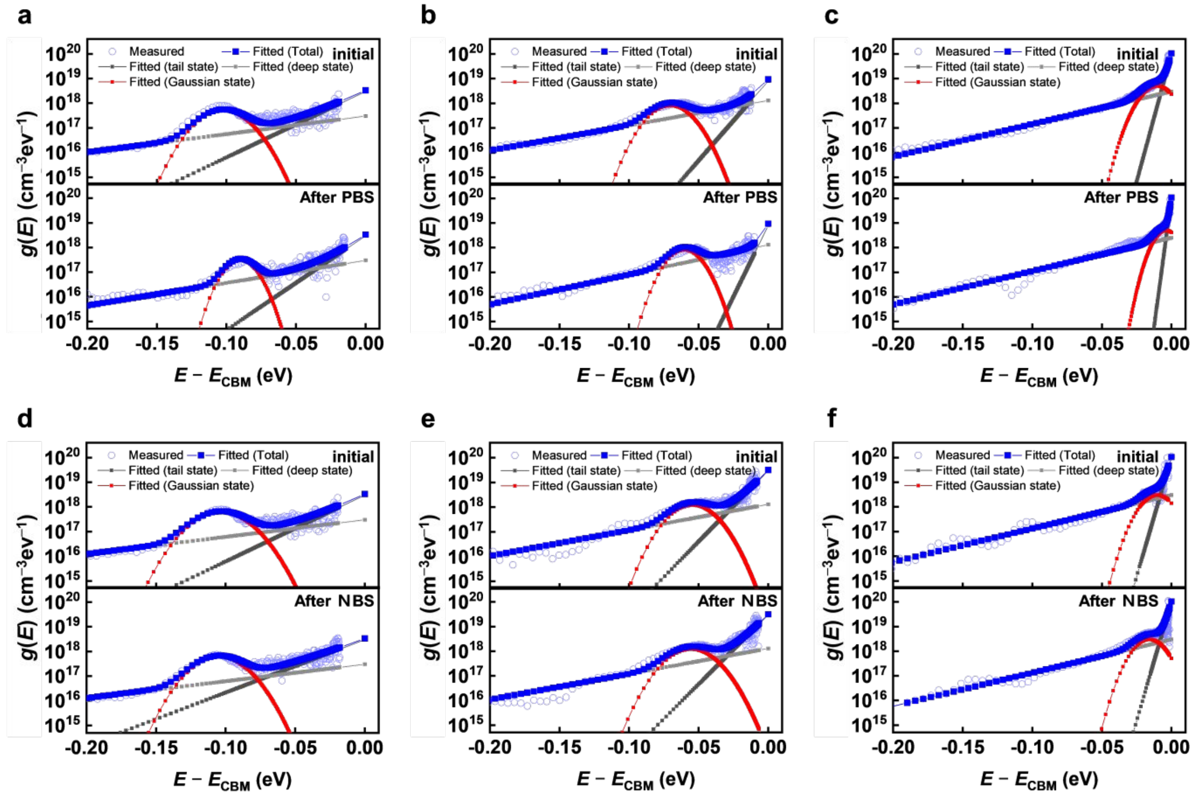

Figure S8. Measured and fitted photo-response C–V results of IGO TFTs with compositions of 6:3, 9:3, and 12:3. DOS changes of the corresponding devices after PBS (a–c) and NBS (d–f).

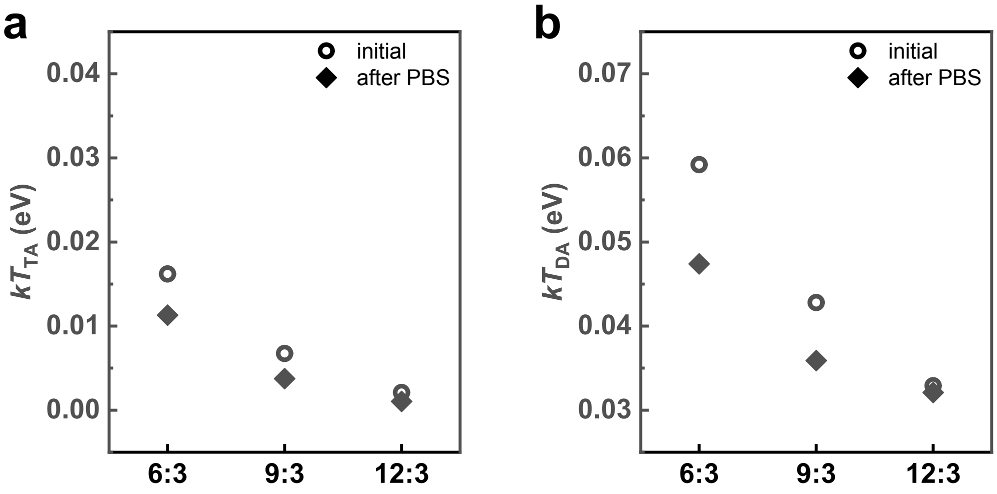

Figure S9. Extracted characteristic energy width (kT) of (a) acceptor-like tail states and (b) acceptor-like deep states before and after PBS for each of the three devices.

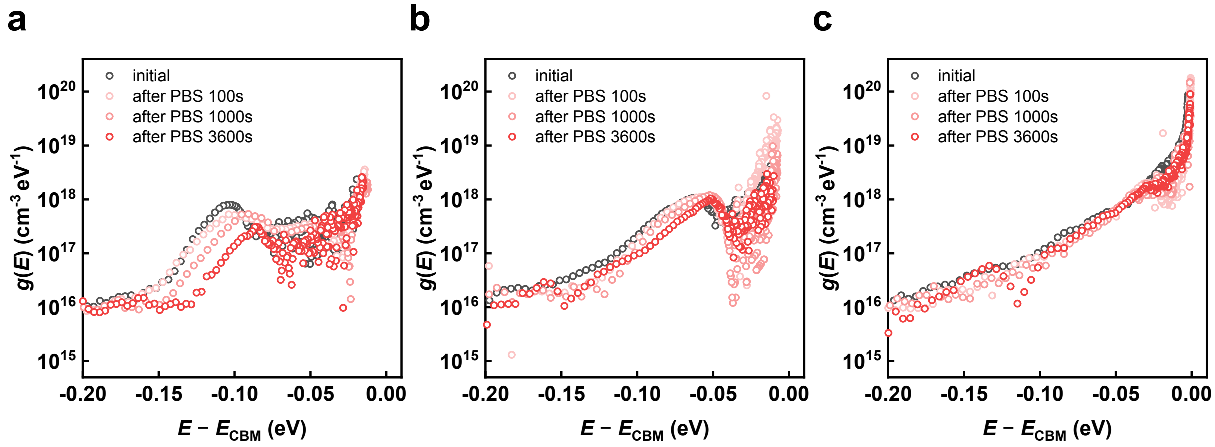

Figure S10. Time-dependent TDOS of IGO TFTs with compositions of (a) 6:3, (b) 9:3, and (c) 12:3 under positive bias stress, obtained using photo-response C–V.

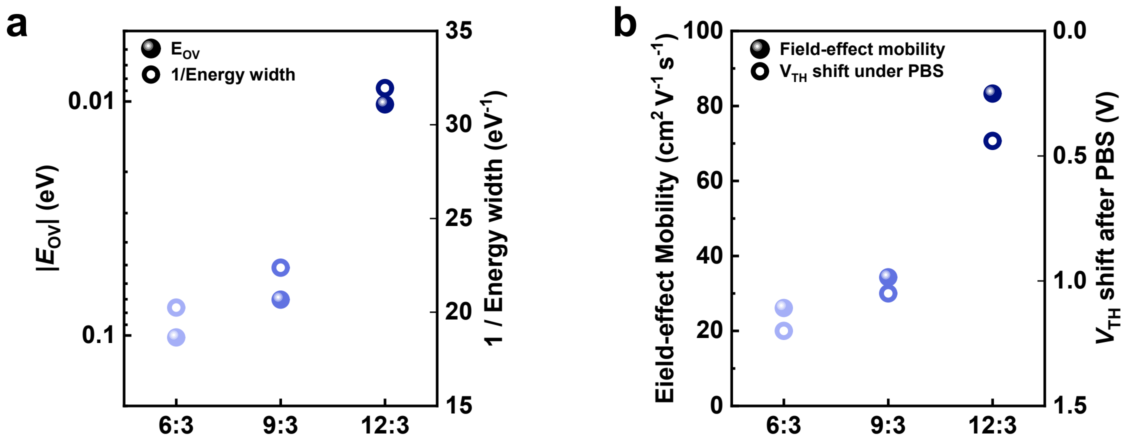

Figure S11. (a) Peak position of the Gaussian state ($|E_{ov}|$) and inverse energy width of total near-CBM states for each of the three devices. The energy axis is referenced to the conduction band minimum (CBM), such that $E_{OV} = 0$ eV corresponds to the CBM. A higher 1/energy width indicates a steeper exponential slope. (b) Field-effect mobility and V_{TH} shift after PBS for each of the three devices.

Table S1. Extracted acceptor-like trap model parameters for each device before and after PBS.

Sample	IGO (6:3)		IGO (9:3)		IGO (12:3)	
	initial	after PBS	initial	after PBS	initial	after PBS
N_{TA} (cm^{-3} eV^{-1})	3.00×10^{18}	3.00×10^{18}	8.00×10^{18}	8.00×10^{18}	1.00×10^{20}	1.00×10^{20}
kT_{TA} (eV)	1.62×10^{-2}	1.13×10^{-2}	6.74×10^{-3}	3.75×10^{-3}	2.10×10^{-3}	1.04×10^{-3}
N_{DA} (cm^{-3} eV^{-1})	3.00×10^{17}	3.00×10^{17}	1.30×10^{18}	1.30×10^{18}	3.00×10^{18}	2.50×10^{18}
kT_{DA} (eV)	5.92×10^{-2}	4.74×10^{-2}	4.28×10^{-2}	3.59×10^{-2}	3.29×10^{-2}	3.21×10^{-2}
N_{OV} (cm^{-3} eV^{-1})	5.00×10^{17}	3.00×10^{17}	8.00×10^{17}	8.00×10^{17}	5.00×10^{18}	5.00×10^{18}
E_{OV} (eV)	-1.02×10^{-1}	-8.99×10^{-2}	-7.03×10^{-2}	-6.01×10^{-2}	-1.03×10^{-2}	-4.00×10^{-3}
kT_{OV} (eV)	1.81×10^{-2}	1.18×10^{-2}	1.55×10^{-2}	1.25×10^{-2}	1.18×10^{-2}	8.81×10^{-3}

Table S2. Extracted acceptor-like trap model parameters for each device before and after NBS.

Sample	IGO (6:3)		IGO (9:3)		IGO (12:3)	
	initial	after PBS	initial	after PBS	initial	after PBS
N_{TA} (cm^{-3} eV^{-1})	3.00×10^{18}	3.00×10^{18}	3.00×10^{19}	3.00×10^{19}	1.00×10^{20}	1.00×10^{20}
kT_{TA} (eV)	1.61×10^{-2}	2.02×10^{-2}	7.56×10^{-3}	7.71×10^{-3}	2.25×10^{-3}	2.25×10^{-3}
N_{DA} (cm^{-3} eV^{-1})	3.00×10^{17}	3.00×10^{17}	1.30×10^{18}	1.30×10^{18}	3.00×10^{18}	3.00×10^{18}
kT_{DA} (eV)	6.25×10^{-2}	6.32×10^{-2}	4.15×10^{-2}	4.18×10^{-2}	3.20×10^{-2}	3.21×10^{-2}
N_{OV} (cm^{-3} eV^{-1})	6.00×10^{17}	6.00×10^{17}	1.20×10^{17}	1.20×10^{17}	3.00×10^{18}	3.00×10^{18}
E_{OV} (eV)	-1.03×10^{-1}	-1.05×10^{-1}	-5.43×10^{-2}	-5.53×10^{-2}	-1.03×10^{-2}	-1.58×10^{-2}
kT_{OV} (eV)	2.05×10^{-2}	1.91×10^{-2}	1.65×10^{-2}	1.78×10^{-2}	1.19×10^{-2}	1.19×10^{-2}

Table S3. Summary of key device performance metrics of optimized crystalline IGO TFTs

Key performance indicator	This work (IGO 12:3)
Channel structure	Zn-free crystalline IGO TFT
Channel thickness	5 nm
Peak mobility (μ_{FE})	83.3 cm^2 V^{-1} s^{-1}
Off-current	~10^{-13} A
ΔV_{TH} (PBS/NBS)	< 0.5 V / < 0.1 V
Reproducibility	20 devices, 2 batches
Inverter gain	647

Supplementary Note 1. Role and control of oxygen-vacancy-related trap states in thin-channel oxide TFTs

In oxide semiconductors, reducing the channel thickness has been widely adopted as an effective strategy to improve device performance by enhancing electrostatic gate control. Compared to bulk oxide FETs, thin-channel TFTs exhibit improved subthreshold characteristics and reduced threshold-voltage variation due to more uniform gate-field penetration across the channel. In addition, confining carrier transport to a narrow channel region suppresses the influence of bulk trap states and spatially inhomogeneous percolation paths, resulting in more reproducible mobility and bias stability. Under such thin-channel conditions, carrier transport becomes increasingly sensitive to interface and near-interface trap states.

In this context, oxygen-vacancy-related states are known to play a dual role in oxide semiconductors, depending on their energetic position. When oxygen vacancies introduce shallow states near the conduction band minimum, they act as donor-like states that enhance electron accumulation and electrical conductivity, thereby contributing to increased field-effect mobility, as widely reported in oxide TFTs including In_2O_3-based systems.[1] In contrast, when oxygen-vacancy-related states are energetically deeper or excessively abundant, they act as carrier trapping and scattering centers, leading to degraded carrier transport, reduced mobility, and poorer electrical stability.[2] These contrasting roles indicate that device performance is governed not simply by the presence of oxygen vacancies, but by their energetic distribution and effective density. Previous studies have shown that the distribution and activity of oxygen-vacancy-related states can be systematically controlled through materials processing. Representative approaches include the use of vacancy suppressors or dopants to passivate defect states,[3,4] regulation of oxygen content during channel-layer deposition to tune vacancy formation,[5] and post-deposition thermal annealing to reduce interface trap densities and relax defect configurations.[6] Collectively, these results highlight that controlling oxygen-vacancy energetics, rather than completely eliminating vacancies, is essential for optimizing transport properties and bias stability in thin-channel oxide TFTs.

Supplementary Note 2. Experimental parameters and methodological considerations

PECCS Measurements

The optical power density used in the PECCS measurements was calibrated using a power meter to 5.714 mW cm^{-2} (0.865 μW over 14,000 μm^2). This corresponds to a photon flux exceeding 1×10^{16} photons cm^{-2} s^{-1}, which is several orders of magnitude higher than the estimated trap density (~10^{12} cm^{-2}).[7] Accordingly, the PECCS response is governed by trap occupancy rather than photon availability. To ensure a well-defined initial trap occupancy, the gate voltage was swept from positive to negative bias, initializing an electron-accumulation state in the n-type oxide channel and filling electron trap states prior to photo-excitation. This sweep configuration enables selective evaluation of photo-driven V_{TH} shifts associated with electron detrapping. In contrast, sweeping from negative to positive bias under illumination can induce measurement error due to negative-bias illumination stress, where the combined electric field and optical excitation activate additional trap states beyond the intended PECCS energy window, leading to extrinsic V_{TH} shifts. Under this measurement scheme, the PECCS measurements were conducted by sweeping the gate voltage from +10 to −10 V with a step of 0.05 V and a dwell time of 0.1 s, corresponding to an effective scan rate of 0.5 V s^{-1}. Importantly, no meaningful change in the PECCS response was observed upon increasing the scan rate, indicating that the extracted results are not dominated by scan-rate-dependent artifacts within the measurement window.

Photo-response C–V Measurements

For photo-response C–V measurements, a 450 nm laser diode with an optical power density of 6.369 mW cm^{-2} was used as the light source. This illumination level was chosen to ensure a photon flux sufficient to avoid photon-limited response, while suppressing excessive photo-stressing effects such as persistent photoconductivity, thermal drift, and baseline instability that can arise at higher optical intensities. To probe photo-induced capacitance variations associated with trap-state charging, the gate voltage was swept from +15 to −15 V with a step of 0.05 V. At each bias point, the capacitance was measured by applying a small-signal AC excitation, with a per-step acquisition time of 0.5 s to allow adequate signal settling and to minimize point-to-point scatter. An AC excitation amplitude of 30 mV was employed to satisfy the small-signal condition while maintaining sufficient signal-to-noise ratio. Much larger excitation amplitudes can induce

nonlinear averaging and measurement-assisted trapping or detrapping, whereas smaller amplitudes degrade capacitance resolution and increase noise. All photo-response C–V measurements were performed at a frequency of 100 kHz, which is sufficiently high to suppress low-frequency dispersion and slow border-trap contributions, while avoiding high-frequency artifacts associated with series resistance and parasitic impedance.[8]

Supplementary Note 3. Methodological scope of optoelectronic characterizations and limitations

The optoelectronic characterization employed in this study is designed to probe trap states that actively contribute to bias instability under photo-bias operation. Under room-temperature and dark conditions, trap states with long characteristic time constants often evolve too slowly to be directly captured within practical measurement times. By simultaneously applying optical excitation and gate bias, carrier generation and electrostatic driving forces are enhanced, which accelerates charge capture and emission processes of trap states that can be activated optically and electrically. This allows sensitive detection of TDOS variations that are directly relevant to photo-response and bias-stress stability and are difficult to access using purely electrical or frequency-domain techniques under dark, small-signal conditions.[9,10]

From a bias-stability standpoint, this approach provides a complementary capability compared with conventional multi-frequency C–V measurements[9] and model-based full sub-bandgap TDOS reconstruction methods.[10] Multi-frequency C–V analysis is effective for resolving trap responses through frequency dispersion but primarily probes trap states under equilibrium electrical conditions. Model-based TDOS reconstruction enables a comprehensive description of the sub-bandgap states but does not specifically target trap activation under photo-bias stress. In contrast, the present optoelectronic approach selectively probes trap states that become active under the applied photo-bias stress conditions and directly influence bias instability.

Several methodological limitations should also be noted. The present analysis does not resolve trap states according to their individual time constants, nor does it uniquely separate donor-like, acceptor-like, and interface trap states across the entire sub-bandgap. Therefore, the extracted TDOS should be interpreted as an effective density of trap states that are activated under photo-bias stress, rather than the total trap density present in the device. Importantly, this effective TDOS corresponds to the subset of trap states that governs bias-stress-induced electrical instability under operating conditions relevant to optoelectronic devices.

REFERENCES

1. Avis, C.; Jang, J. In_2O_3: An Oxide Semiconductor for Thin-Film Transistors, a Short Review. *Molecules* 2025, **30**, 4762. DOI10.3390/molecules30244762

2. Park, B. Modulating the Electrical Properties of Lithium-Doped Zinc Oxide: Interplay of Lithium Content, Oxygen Pressure, and Temperature. *Korean J. Met. Mater.* 2024, **62**, 106–114. DOI: 10.3365/KJMM.2024.62.2.106

3. Lee, S.; Kim, S. J.; Lee, S. Y. Role of Various Oxygen Vacancy Suppressors on the Electrical Performance of Amorphous Zn–Sn–O Thin-Film Transistors. *J. Mater. Sci.: Mater. Electron.* 2025, 2299. DOI:10.1007/s10854-025-16323-4

4. Kim, H. G.; Lee, H. J.; Lee, K. M.; Kim, T. G. Improved Mobility and Bias Stability of Hf-Doped IGZO/IZO/Hf-Doped IGZO Thin-Film Transistor. *J. Alloys Compd.* 2024, **981**, 173587. DOI: 10.1016/j.jallcom.2024.173587

5. Jeong, H.-S.; Cha, H.-S.; Hwang, S.-H.; Lee, D.-H.; Song, S.-H.; Kwon, H.-I. Effects of Oxygen Content on Operational Characteristics and Stability of High-Mobility IGTO Thin-Film Transistors during Channel Layer Deposition. *Coatings* 2021, **11**, 698. DOI: 10.3390/coatings11060698.

6. Trinh, T. T.; Nguyen, V. D.; Ryu, K.; Jang, K.; Lee, W.; Baek, S.; Raja, J.; Yi, J. Improvement in the Performance of an InGaZnO Thin-Film Transistor by Controlling Interface Trap Densities between the Insulator and Active Layer. *Semicond. Sci. Technol.* 2011, **26**, 085012. DOI: 10.1088/0268-1242/26/8/085012

7. Lee, K.; Oh, M. S.; Mun, S. J.; Lee, K. H.; Ha, T. W.; Kim, J. H.; Park, S. H. K.; Hwang, C. S.; Lee, B. H.; Sung, M. M.; Im, S. Interfacial Trap Density-of-States in Pentacene- and ZnO-Based Thin-Film Transistors Measured via Novel Photo-Excited Charge-Collection Spectroscopy. *Adv. Mater.* 2010, **22**, 3260–3265. DOI: 10.1002/adma.201000722

8. Jeon, K.; Kim, C.; Song, I.; Park, J.; Kim, S.; Kim, S.; Park, Y.; Park, J.-H.; Lee, S.; Kim, D. M.; Kim, D. H. Modeling of Amorphous InGaZnO TFTs Based on DOS Extracted from Optical Response of C–V Characteristics. *Appl. Phys. Lett.* 2008, **93**, 182102. DOI: 10.1063/1.3013842

9. Lee, S.; Park, S.; Kim, S.; Jeon, Y.; Jeon, K.; Park, J.-H.; Park, J.; Song, I.; Kim, C. J.; Park, Y.; et al. Extraction of Subgap Density of States in Amorphous InGaZnO Thin-Film Transistors by Using Multifrequency Capacitance–Voltage Characteristics. *IEEE Electron Device Lett.* 2010, **31** (3), 231–233. DOI: 10.1109/LED.2009.2039634

10. Kim, Y.; Bae, M.; Kim, W.; Kong, D.; Jeong, H. K.; Kim, H.; Choi, S.; Kim, D. M.; Kim, D. H. Amorphous InGaZnO Thin-Film Transistors—Part I: Complete Extraction of Density of States Over the Full Subband-Gap Energy Range. *IEEE Trans. Electron Devices* 2012, **59** (10), 2689–2698. DOI: 10.1109/TED.2012.2208969
